# Insights into the dual cleavage activity of the GH16 laminarinase enzyme class on β-1,3 and β-1,4 glycosidic bonds

**DOI:** 10.1016/j.jbc.2021.100385

**Published:** 2021-02-05

**Authors:** Marcelo Vizona Liberato, Erica Teixeira Prates, Thiago Augusto Gonçalves, Amanda Bernardes, Nathalia Vilela, Juliana Fattori, Gabriela Cristina Ematsu, Mariana Chinaglia, Emerson Rodrigo Machi Gomes, Ana Carolina Migliorini Figueira, André Damasio, Igor Polikarpov, Munir S. Skaf, Fabio Marcio Squina

**Affiliations:** 1 Laboratório Nacional de Ciência e Tecnologia do Bioetanol (CTBE), Centro Nacional de Pesquisa em Energia e Materiais (CNPEM), Campinas, São Paulo, Brazil; 2 Programa de Processos Tecnológicos e Ambientais, Universidade de Sorocaba, Sorocaba, São Paulo, Brazil; 3 Instituto de Química e Centro de Pesquisa em Engenharia e Ciências Computacionais, Universidade Estadual de Campinas, Campinas, São Paulo, Brazil; 4 Departamento de Bioquímica e Biologia Tecidual, Instituto de Biologia, Universidade de Campinas, Campinas, São Paulo, Brazil; 5 Instituto de Física de São Carlos, Universidade de São Paulo, São Carlos, São Paulo, Brazil; 6 Laboratório Nacional de Biociências (LNBio), Centro Nacional de Pesquisa em Energia e Materiais (CNPEM), Campinas, São Paulo, Brazil

**Keywords:** glycoside hydrolase, transglycosylation, GH16, laminarinase, endo-1,3(4)-β-glucanase, metagenome, BGB, 1,3-β-D-cellotriosyl-glucose, BGC, 1,3-β-D-cellobiosyl-cellobiose, C2, cellobiose, C3, cellotriose, C4, celloheptaose, C5, cellopentaose, C6, cellohexaose, DP, degree of polymerization, G3, 1,3-β-D-cellobiosyl-glucose, GH, glycoside hydrolases, ITC, isothermal titration calorimetry, LamR, laminarinase from *Rhodothermus marinus*, L2, laminaribiose, L3, laminaritriose, L5, laminaripentaose, L4, laminariheptaose, L6, laminarihexaose, MD, molecular dynamics, MR, molecular replacement, PDB, Protein Data Bank, SCLam, a GH16 member derived from a soil metagenome, TpLam, laminarinase from *Thermotoga petrophila*

## Abstract

Glycoside hydrolases (GHs) are involved in the degradation of a wide diversity of carbohydrates and present several biotechnological applications. Many GH families are composed of enzymes with a single well-defined specificity. In contrast, enzymes from the GH16 family can act on a range of different polysaccharides, including β-glucans and galactans. SCLam, a GH16 member derived from a soil metagenome, an endo-β-1,3(4)-glucanase (EC 3.2.1.6), can cleave both β-1,3 and β-1,4 glycosidic bonds in glucans, such as laminarin, barley β-glucan, and cello-oligosaccharides. A similar cleavage pattern was previously reported for other GH16 family members. However, the molecular mechanisms for this dual cleavage activity on (1,3)- and (1,4)-β-D-glycosidic bonds by laminarinases have not been elucidated. In this sense, we determined the X-ray structure of a presumably inactive form of SCLam cocrystallized with different oligosaccharides. The solved structures revealed general bound products that are formed owing to residual activities of hydrolysis and transglycosylation. Biochemical and biophysical analyses and molecular dynamics simulations help to rationalize differences in activity toward different substrates. Our results depicted a bulky aromatic residue near the catalytic site critical to select the preferable configuration of glycosidic bonds in the binding cleft. Altogether, these data contribute to understanding the structural basis of recognition and hydrolysis of β-1,3 and β-1,4 glycosidic linkages of the laminarinase enzyme class, which is valuable for future studies on the GH16 family members and applications related to biomass conversion into feedstocks and bioproducts.

The plant cell wall is mainly composed of cellulose and hemicellulose, which are the most abundant biopolymers on Earth. Glycoside hydrolases (GHs) are major enzymes involved in the breakdown of plant cell wall carbohydrates (1, 2). Owing to the recalcitrance and variation in composition of different plants, tissues, stage, and growth conditions, a wide variety of GHs are required for plant cell wall deconstruction (3, 4). The enzymatic conversion of plant cell wall polysaccharides into fermentable monosaccharides has been extensively studied to produce second-generation biofuels and other chemicals. Furthermore, chemical synthesis of specific oligosaccharides is extremely difficult but can potentially be done with enzymatic technologies (5).

GHs typically display hydrolytic activity, where the glycosidic bond is cleaved using water by either a single (inverting) or double (retaining) displacement mechanism. The retaining hydrolysis mechanism occurs in two steps: first, known as glycosylation, when the glycosidic bond is broken to form the covalent glycosyl-enzyme intermediate. This step is followed by a second, deglycosylation, step, in which a water molecule acts as an acceptor cleaving the glycosyl-enzyme intermediate through hydrolysis (6). Alternatively, transglycosylation may occur when an oligosaccharide is used as an acceptor during the deglycosylation step, instead of a water molecule (5, 7).

Among GH families, GH16 is a highly diversified one that has recently been divided into 23 subgroups based on Sequence Similarity Network analysis (8). Despite the similar β-jelly roll folding shared by all GH16 members, each group may have a specific or broad range of functions, including xyloglucanase (EC 3.2.1.151), β-agarase (EC 3.2.1.81), κ-carrageenase (EC 3.2.1.83), endo-β-1,3-galactanase (EC 3.2.1.), β-porphyranase (EC 3.2.1.178), endo-1,3-β-glucanase (EC 3.2.1.39), (1,3,1,4)-β-D-glucan endohydrolases (EC 3.2.1.73), and (1,3/1,3;1,4)-β-D-glucan endohydrolases (EC 3.2.1.6) (9). Both (1,3,1,4)-β-D-glucan endohydrolases and (1,3/1,3;1,4)-β-D-glucan endohydrolases act on degradation of mixed-linkage β-glucans, which are found in nearly all members of the Poaceae family, including grasses and cereals (10). The main difference between these two subgroups is that, (1,3,1,4)-β-D-glucan endohydrolases hydrolyze only (1,4)-β-D-glycosyl bonds, whereas (1,3/1,3;1,4)-β-D-glucan endohydrolases are able to cleave both β-1,3 and β-1,4 bonds (11). According to previous publications, in both cases the presence of a (1,3)-β-D-glycosyl bond connecting the previous glucose unit (at the nonreducing end of the polymer) is considered necessary for correct positioning of the scissile bond within the active site for hydrolysis (11, 12, 13).

Previously we described the identification and biochemical characterization of a (1,3/1,3;1,4)-β-D-glucan endohydrolase derived from the soil metagenome, named as SCLam (14). Of interest, SCLam is able to cleave cellohexaose, which contains only 1,4-β-D-glycosyl linkages. This behavior contradicted the definition of (1,3/1,3;1,4)-β-D-glucan endohydrolase stated above. In 1998, Krah *et al.* reported a similar pattern for the laminarinase LamR from *Rhodothermus marinus* (15). However, the mechanism of this broad specificity has not been elucidated.

In the present study, we carried out a comprehensive biochemical, biophysical, and structural investigation to understand the molecular basis of the cleavage and formation of (1,3)- and (1,4)-β-D-glycosyl bonds by SCLam, via hydrolysis and transglycosylation, respectively. In order to do so, SCLam was crystallized and its inactive mutant form was cocrystallized with different oligosaccharides. The cleavage pattern of SCLam was evaluated with different substrates, such as mixed-linkage (1,3,1,4)-β-D-glucans and mixed linkage gluco- and cello-oligosaccharides. Isothermal titration calorimetry (ITC) analysis with the mutant enzyme was also performed to determine the binding affinities for different oligosaccharides. Finally, molecular dynamic simulations of SCLam complexed with (1,3,1,4)-β-glucopentaose, cellohexaose (C6), and laminarihexaose (L6) provided new insights on the mechanisms of hydrolysis and transglycosylation.

## Results and discussion

### SCLam substrate specificity and hydrolytic degradation pattern

As described previously (14), SCLam degradation of barley β-glucan resulted in accumulation of mainly glucose and oligosaccharides with the degree of polymerization (DP) ranging from two to four glucose units (DP2 to DP4). Barley β-glucan contains a ratio of β-1,4/β-1,3 linkages of ∼2.4 (16) and, thus, the accumulation of glucose indicates that SCLam was able to cleave both β-1,3 and β-1,4 glycosidic bonds. Furthermore, SCLam was able to cleave cellohexaose, confirming its capacity to cleave β-1,4 glycosidic bonds.

**Figure 1:**
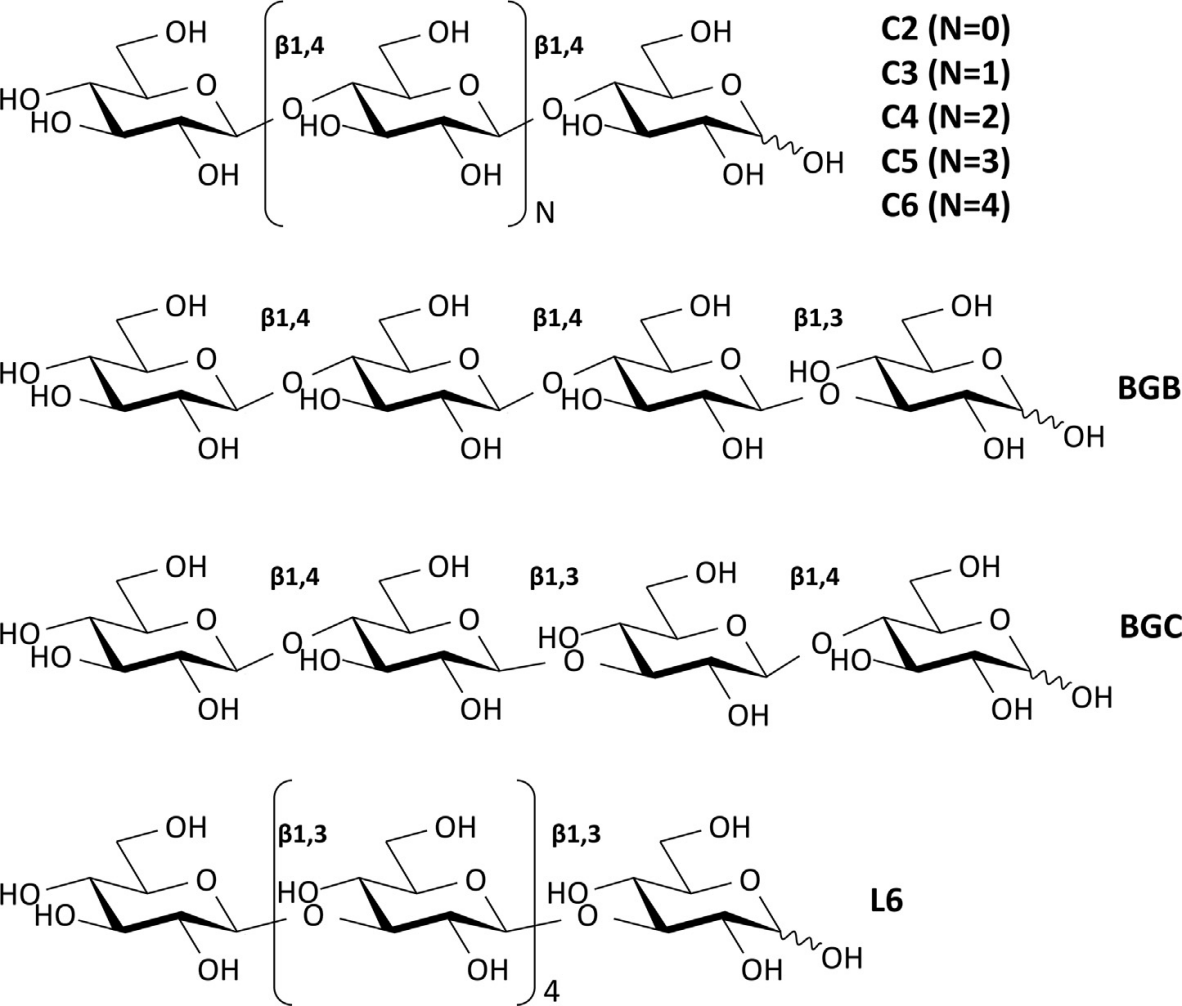
**Gluco-oligosaccharides used in SCLam activity assay**: cellobiose (C2), cellotriose (C3), cellotetraose (C4), cellopentaose (C5), cellohexaose (C6), 1,3-β-D-cellotriosyl-glucose (BGB), 1,3-β-D-cellobiosyl-cellobiose (BGC), and laminarihexaose (L6).

**Figure 2:**
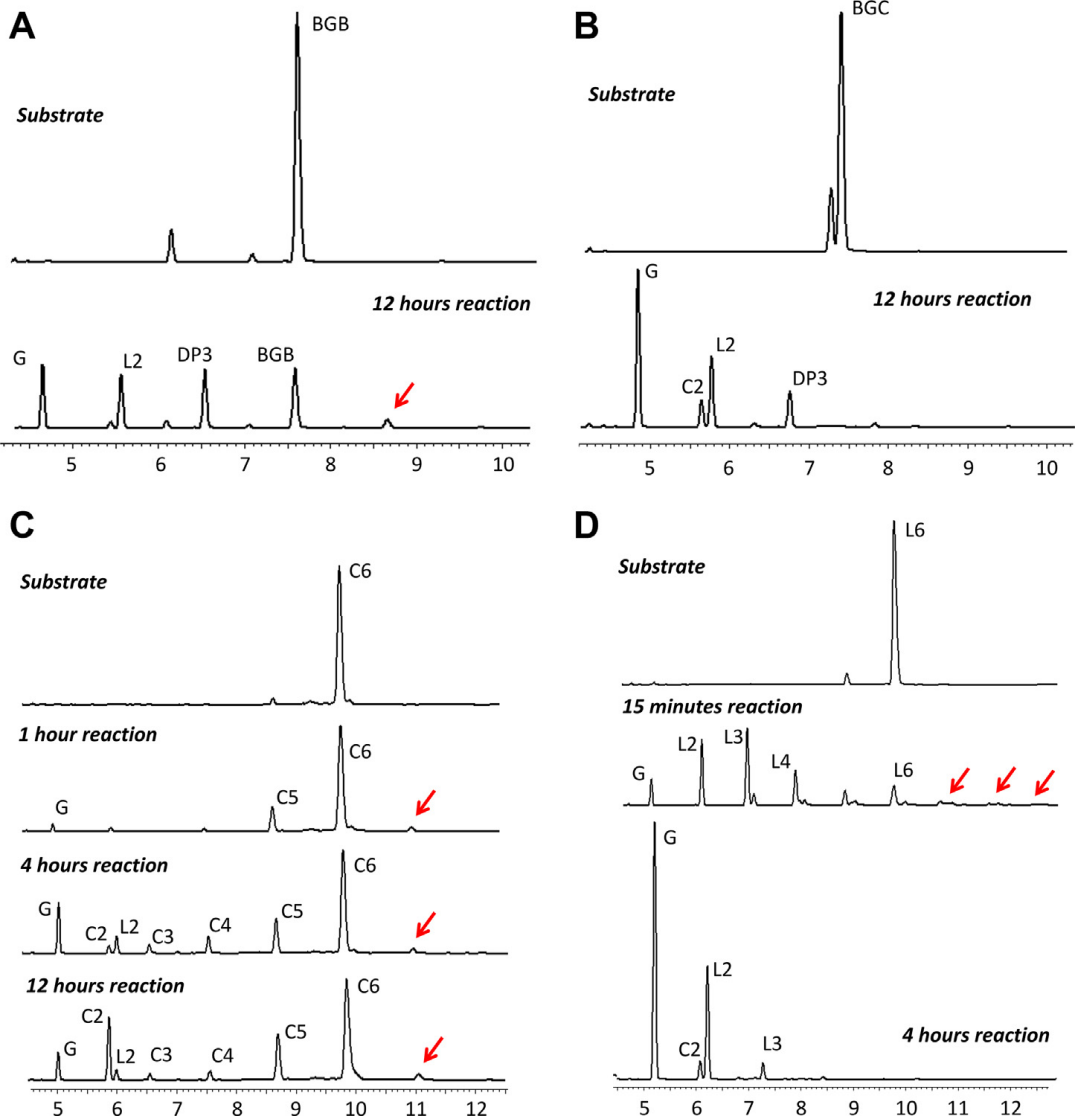
**Evaluation of the SCLam cleavage pattern in different substrates revealed by capillary zone electrophoresis**. Activity against the oligosaccharides BGB (*A*) and BGC (*B*) was tested in 12-h reactions resulting in glucose and oligosaccharides with two and three degrees of polymerization (DP2 and DP3, respectively). Reactions against cellohexaose (*C*) and laminarihexaose (*D*) were performed in different times, resulting in a range of substrates with lower DP down to glucose. The *red arrows* indicate oligosaccharide products with higher degree of polymerization than the substrate, suggesting transglycosylation activity. The double peaks observed between 5.5 and 6.0 min were assigned as Cellobiose and Laminaribiose (see Fig. S6).

To further investigate substrate specificity, SCLam was evaluated in reactions containing a variety of oligosaccharides displaying different proportions and structural organization of β-1,3 and β-1,4 bonds. Initially, SClam activity was evaluated in 1,3-β-D-cellotriosyl-glucose (BGB) and 1,3-β-D-cellobiosyl-cellobiose (BGC), which are gluco-oligosaccharides that contain two β-1,4-bonds and one β-1,3-bond. BGB has the β-1,3-bond on the reducing end and BGC in the middle of the chain (Fig. 1). As shown in Figure 2*B*, after 12 h of incubation, SCLam (4 μM) was able to completely degrade BGC into smaller oligosaccharides and monosaccharides. On the other hand, BGB was only partially degraded by SCLam (Fig. 2*A*). These results reveal that SCLam displays a preference for BGC, which possesses the β-1,3 bond in the middle of the chain, in comparison with BGB that has its β-1,3 bond at the reducing end. Next, the activity of SCLam was tested against cello-oligosaccharides (Figs. 2*C* and S1*A*). As a result, it was possible to observe the formation of oligosaccharide products with lower DP in all cases, including glucose. However, even after 12 h of reaction, the initial substrates could not be fully degraded. Finally, ScLam promoted the complete degradation of L6 as shown in Figure 2*D*.

The degradation pattern of cello-oligosaccharides observed for SCLam was similar to the laminarinase, LamR, from *R. marinus* (15). To evaluate whether the ability to cleave β-1,4 glycosidic bonds could be common to another (1,3/1,3;1,4)-β-D-glucanase from family GH16, we also evaluated the activity of the TpLam from *Thermotoga petrophila* (17) in C5, under the same conditions evaluated for SCLam. As a result, a similar pattern of cleavage was observed (Fig. S1*B*), indicating that this ability can be potentially found in other enzyme members of the subgroup classified as (1,3/1,3;1,4)-β-D-glucan endohydrolases. Collectively, the assays with ScLam (and TpLam) using cellohexaose (and cellopentaose, respectively) as substrate demonstrated the ordered production of C5 (C4) followed by C4 (C3), C3 (C2, respectively), which is expected for typical exo mode of action when cleaving β-1,4 bonds.

**Figure 3:**
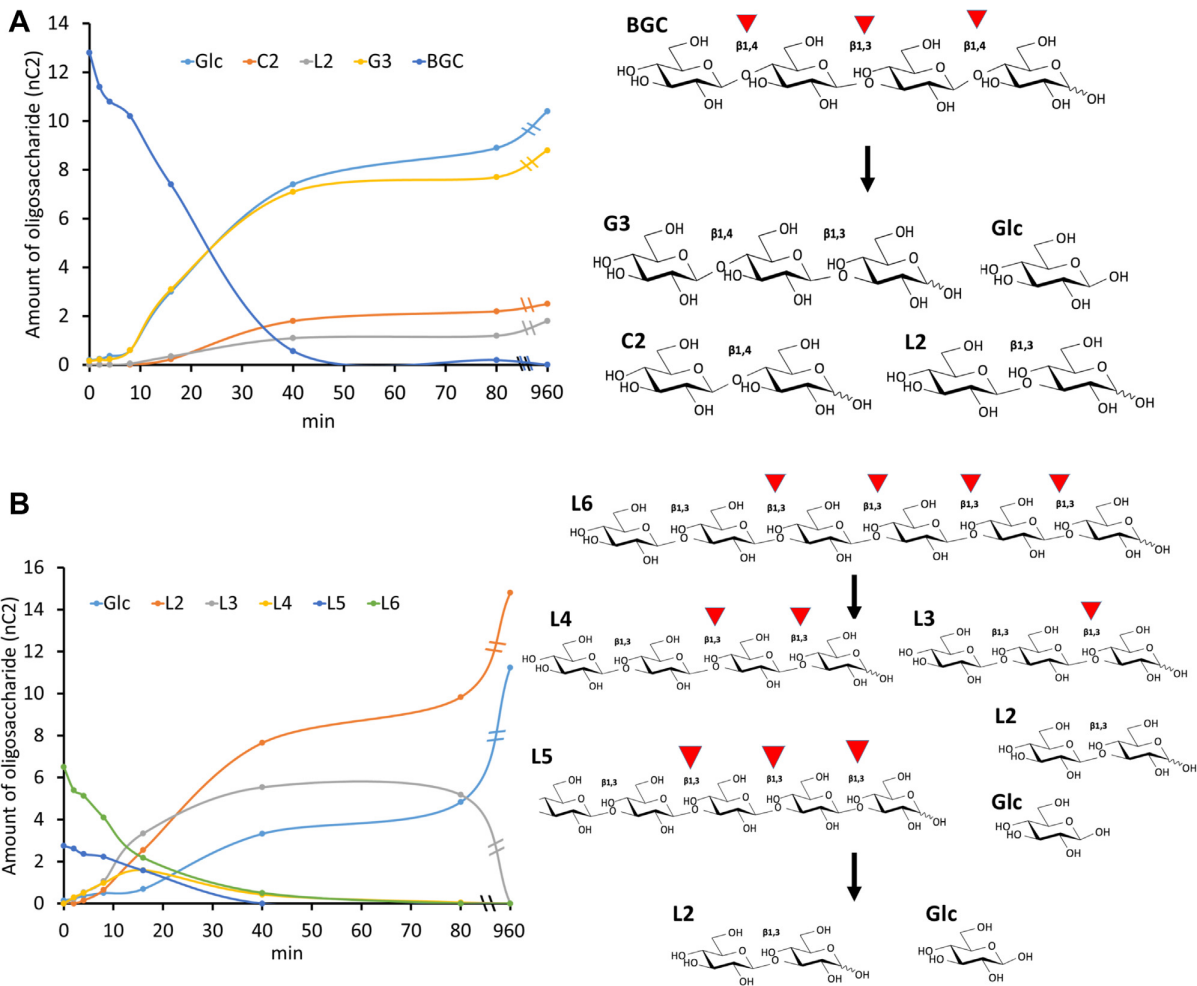
**Activity of SCLam against (*A*) BGC and (B) L6 during time. Both BGC and L6 were completely degraded in about 50-min reactions using SCLam at 5 nM. G3, C2, L2, and glucose (Glc) accumulated as products during BGC hydrolysis, whereas just L2 and Glc resulted from the reaction with L6.** The substrates, products, and subproducts were evaluated with high-performance anion exchange chromatography with pulsed amperometric detection. The amount of each oligosaccharide was plotted as peak areas (the original high-performance anion exchange chromatography with pulsed amperometric detection graphs can be found in Fig. S2). The probable reaction models are demonstrated at the right side of each graph and cleavage sites were depicted with *arrowheads*.

The assays demonstrated that SCLam fully cleaves L6 and BGC (Figs. 2 and S3), which present a β-1,3 linkage next to the scissile bond toward the nonreducing end (Fig. 1). Enzymatic reactions (5 nM SCLam) containing L6 and BGC were further monitored by ion chromatography to provide additional insights into the SCLam mode of operation (Figs. 3 and S2). The results indicated a very similar activity rate with L6 and BGC, where both substrates were entirely degraded in about 50 min. However, the L6 products (L5, L4, and L3) may impact the competition for the binding site. In the case of BGC, the primary degradation products are not further degraded. Considering the initial step of the curves (linear degradation phase), SCLam degraded L6 and BGC at rates 2.3 and 1.3 nmol/min, respectively. Therefore, SCLam is slightly more active when cleaving L6 than BGC.

### SCLam transglycosylates substrates

As indicated by red arrows in Figure 2 (more evidently represented in Fig. S3), the appearance of peaks corresponding to products of high DP for all evaluated substrates suggests the occurrence of transglycosylation activity, which is a property related to retaining hydrolases (18), such as GH16 family members (19, 20).

Moreover, SCLam^E144S^ presented evident transglycosylation activity toward all tested substrates, concomitantly with a substantial reduction in hydrolytic activity. As previously described, the substitution of the nucleophile by serine or alanine leads to a decrease in glucosidase activity (21). Of interest, the mutation of these residues in the sucrose-binding region of invertases also enhances transglycosylation activity (5). The putative transglycosylation also explains the appearance of L2 and C2 as products of the cleavage of C6 and L6, respectively (Figs. 2 and 3).

### Binding affinity of the mutant SCLam^E144S^ for oligosaccharides

The binding affinity of SCLam for different oligosaccharides was assessed through ITC, using the inactive mutant SCLam^E144S^ (catalytic nucleophile mutation). The complete description of ITC results is shown in Table 1 and the corresponding graphs are displayed in Figure S4.

**Table 1:** Thermodynamic parameters calculated from ITC for SCLam^E144S^ interaction with different oligosaccharides

Ligand	*K*_a_ × 10^4^ (M^−1^)	Δ*G* (kcal mol^−1^)	Δ*H* (kcal mol^−1^)	*T*Δ*S* (kcal mol^−1^)	*n*
BGB	66.7 (±1.4)	−7.81	−16.10 (±0.03)	−8.29	1.2 (±0.01)
L6 site 1	17.8 (±2.2)	−6.93	−17.80 (±0.11)	−10.87	
C6 site 1	9.7 (±0.56)	−6.69	−6.54 (±0.06)	0.15	
BGC	0.59 (±0.07)	−5.08	−11.5 (±4.29)	−6.42	1.21 (±0.42)
L6 site 2	ND	ND	ND	ND	
C6 site 2	ND	ND	ND	ND	
C4	ND	ND	ND	ND	ND

ND, not determined.

The parameters for BGB and BGC curves were calculated using single-site binding models, while for L6 and C6 a sequential binding model was applied. For L6 and C6 site 2, the parameters could not be calculated with reasonable errors, as well as for C4. As revealed by crystallographic structures, site 1 corresponds to the catalytic negative subsites and site 2, to the positive subsites.

ITC curves of SCLam^E144S^ to BGB and BGC ligands were fitted with single-site binding models. However, a sequential binding model had to be used to properly fit the L6 binding curve. For C6, both models generated a reasonable fit (Fig. S4), but the statistics from the single-site binding model resulted in n = 1.81, indicating a second site. Hence, the sequential binding model was also used for C6.

All interactions resulted from favorable enthalpy, but a variation in entropy was observed among the ligands, which is correlated to the presence or absence of the β-1,3 glycosidic bond. As shown in Table 1, interactions of SCLam^E144S^ with BGB, L6, and BGC, all presenting β-1,3 linkages, led to a considerable reduction in entropy. By contrast, the thermodynamics of binding to C6 is defined by a lower enthalpic contribution, with a small, but positive, entropic component.

The ligands composed of a β-1,3 linkage at the reducing end (BGB and L6) showed higher affinity for SCLam^E144S^ compared with BGC, C6, and C4. The highest affinity for SCLam^E144S^ was observed for BGB (*K*_a_ = 66.7 × 10^4^ M^−1^) followed by L6 (*K*_a_ = 17.8 × 10^4^ M^−1^), C6 (*K*_a_ = 9.7 × 10^4^ M^−1^), and BGC (*K*_a_ = 0.6 × 10^4^ M^−1^). C4 ligand displayed very low affinity for SCLam^E144S^, and the data could not be fitted to a binding model.

According to activity data, it was expected that the β-1,3-containing substrates (BGB and L6) would have higher affinity to SCLam^E144S^ in comparison with cello-oligosaccharides. The SCLam^E144S^ showed a 100-fold higher affinity to BGB than to BGC. This result is in contrast to the activity assays where the enzyme displays stronger preference for BGC over BGB (Fig. 2). The reason for this apparent contradiction can be explained by the crystallographic structures, which showed a trisaccharide with β-1,3 linkage at the reducing end bound to only negative subsites, a position not suitable for cleavage (discussed below). In summary, BGB may have high affinity to bind into negative subsites, thus preventing cleavage, whereas the BGC structure leads to optimal binding for hydrolysis.

### SCLam crystallographic model and interaction of SCLam^E144S^ with ligands

The crystallographic structures of SCLam and SCLam^E144S^ were determined at resolutions varying from 1.5 to 2.2 Å. Statistics from data collection and refinement are given in Table S1. All structures have a monomer in the asymmetric unit, and all amino acids from SCLam were built in the final models. The amino acid residues of the SCLam^E144S^ models that were not built owing to poor electron density are shown in Table S2.

The six models generated, including the wildtype and ligand-bound mutant, were nearly identical. Their overlap resulted in an RMSD of 0.228 Å, and all amino acid residues in the binding site have the same positions and orientations. The unique difference is the presence of a glycerol molecule, modeled in three alternative conformations, at subsite -1 and the position of the N-terminal loop in SCLam. Owing to the absence of ligands, the N-terminal region in SCLam is fitted to one end of the binding cleft, with the residue M1 occupying the subsite +2 and part of +1 (Fig. S5). In contrast, the N-terminal regions in all SCLam^E144S^ structures are solvent exposed and exhibit multiple conformations.

**Figure 4:**
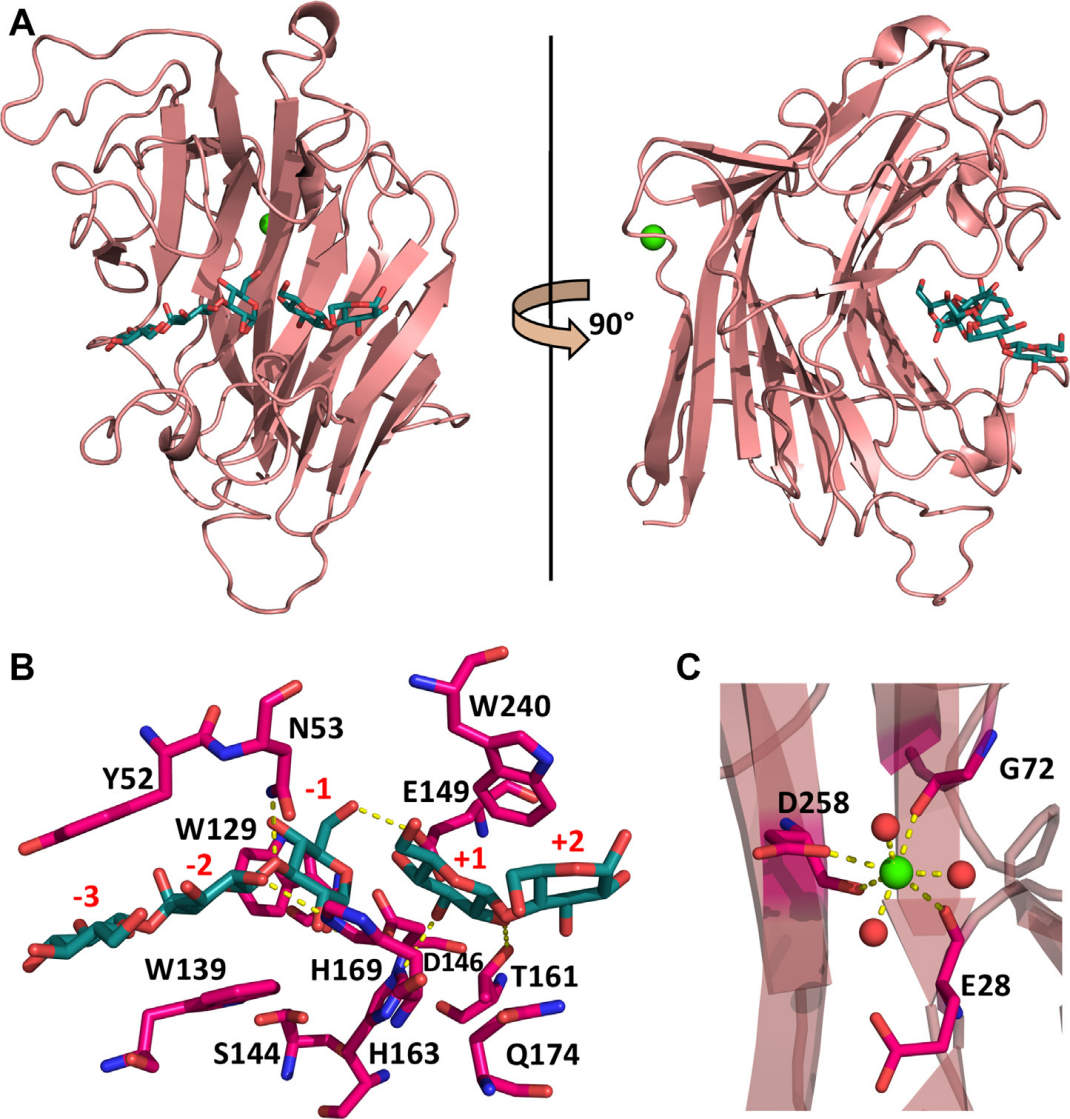
**Crystallographic model of SCLam**^**E144S**^**cocrystallized with C3**. *A*, SCLam has a β-jelly-roll folding, with the substrate-binding site located at the concave face and the calcium-binding site at the convex face. *B*, the ligands G3 (from the cocrystallization with C3), in *blue*, are coordinated by hydrogen bonds (*yellow dashes*) with N53, D146, T161, H163, and H169, and CH-π interactions with Y52, W129, W139, and W240. The subsites are labeled in *red*. *C*, the calcium ion (*green sphere*) is heptacoordinated by E28, D258, D258, and three waters (shown as *red spheres*).

The enzyme SCLam has the typical β-jellyroll fold of GH16 family. This fold is characterized by two curved β-sheets with loops connecting the β-strands (Fig. 4*A*). The substrate-binding site is located on the concave face and is composed of the catalytic residues E144 and E149 (defined as nucleophile and acid/base, respectively) as well as other residues responsible for the substrate anchoring (Fig. 4*B*). All structures have a calcium-binding site with a Ca^2+^, heptacoordinated by a hydroxyl group from the E28, G72, and D258 main chain; D258 side chain; and three waters (Fig. 4*C*). The location of the ion was inferred by comparison with similar structures. Although SCLam^E144S^ has been crystallized with 0.1 to 0.2 mM magnesium chloride, the SCLam crystal was obtained in malic acid and all the structures have the same electron density representing the atom. Therefore, the Ca^2+^ probably came from the bacterial host. The calcium-binding site is partially conserved in the GH16 family (22, 23), and its importance for enzyme stability has already been demonstrated (24).

**Figure 5:**
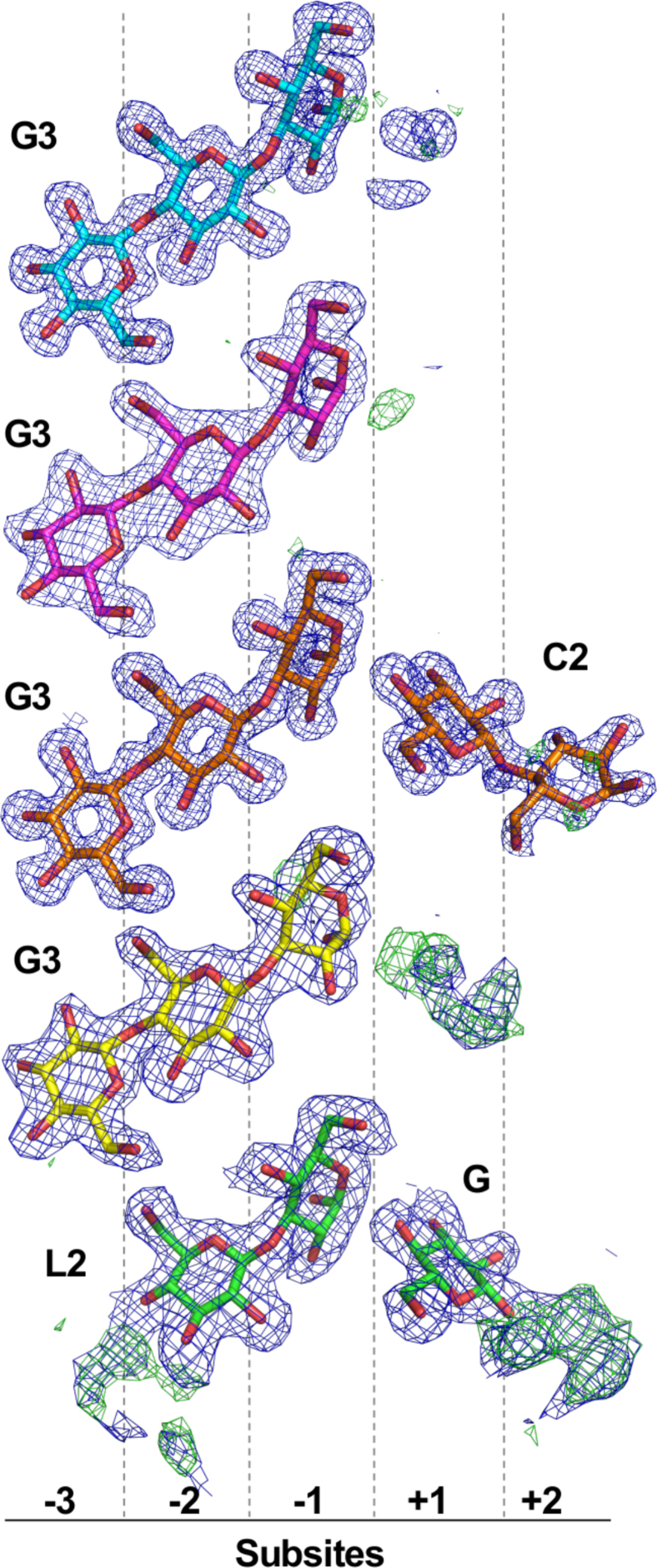
**SCLam**^**E144S**^**was cocrystallized with ligands**, BGB (*blue*), BGC (*magenta*), C3 (*orange*), C6 (*yellow*), and L6 (*green*). However, regardless of the initial compound, the final electron densities (2Fo – Fc, at 1.0 sigma drawn in *blue*) clearly represent 1,3-β-D-cellobiosyl-glucose (G3) in the same orientation, occupying the negative subsites. As an exception, a laminaribiose is clearly seen from cocrystallization with L6, although continuous electron densities from both 2Fo – Fc (1.0 sigma, in *blue*) and Fo – Fc (3.0 sigma, in *green*) maps indicate an oligosaccharide with a higher degree of polymerization. At the positive subsites electron densities were seen, corresponding to a cellobiose (C2) and a glucose (G), when the enzyme was cocrystallized with C3 and L6, respectively. Once again, continuous electron densities indicate the presence of a glucose at +1 subsite of SCLam^E144S^ cocrystallized with C6 and an oligosaccharide with a higher degree of polymerization at positive subsites when cocrystallized with L6.

Although we did not manage to obtain the *apo* form of the mutated enzyme, the structures of SCLam^E144S^ cocrystallized with five different substrates, BGB, BGC, C3, C6, and L6, were solved. It is surprising that, in all structures of enzyme-ligand complexes, except for SCLam^E144S^/L6, clear electron densities confirm the presence of a 1,3-β-D-cellobiosyl-glucose (G3), where the β-1,3 linkage is located at the reducing end, from subsite −3 to −1 (Fig. 5). SCLam^E144S^/L6 reveals a laminaribiose (L2) at subsites −2 and −1, and a continuous electron density from carbon C3 of glucose at subsite −2 suggests that there is at least another glucose monomer, resulting in a laminaritriose (L3) or longer oligosaccharide. This third glucose could not be modeled owing to poor electron density. Considering that C6 and C3 comprise exclusively 1,4 glycosidic bonds, the presence of a glucotriose containing a β-1,3 glycosidic bond can only be explained by substrate cleavage together with transglycosylation activity of the mutant.

The presence of the same ligand (G3) in most structures, even when the enzyme was cocrystallized with cello-oligosaccharides, indicates a high affinity of G3 for this site. It is also noteworthy that all SCLam^E144S^ structures have an α-anomeric glycan in subsite −1. According to Cheng *et al.* (21), although the proportion of α/β anomers in solution is 50:50, the nucleophile mutation to serine can shift its preference toward α configuration.

Considering the positive subsites, SCLam^E144S^/C3 has a cellobiose at subsites +1 and +2. SCLam^E144S^/L6 contains a glucose at subsite +1 and a continuous electron density resembling another glucose at subsite +2, which was not modeled owing to uncertainty about the precise orientation of the ligand in this position. Furthermore, SCLam^E144S^/C6 has a less precise electron density at subsite +1 that could be a glucose. The electron densities found in these subsites are not perfectly defined (Fig. 5). It is therefore possible that more flexible oligosaccharides are occupying the positive subsites. In contrast, SCLam^E144S^/BGB and SCLam^E144S^/BGC do not have a clear electron density at positive subsites. Coincidentally, ITC curves from BGB and BGC were fitted to a single-site binding model. No other undefined electron density was observed in these structures. Thus, we may conclude that the negative subsites could be the "site 1" described based on the L6 and C6 ITC curves and the positive subsites could be the "site 2."

The binding of mono- or oligosaccharides to positive subsites is a critical step for transglycosylation (5). The results from crystallography, ITC, and activity assays evidenced, in concert, this activity. However, there is no evidence regarding why products from C6, C3, and L6 can bind to positive subsites, whereas BGB and BGC products cannot.

**Figure 6:**
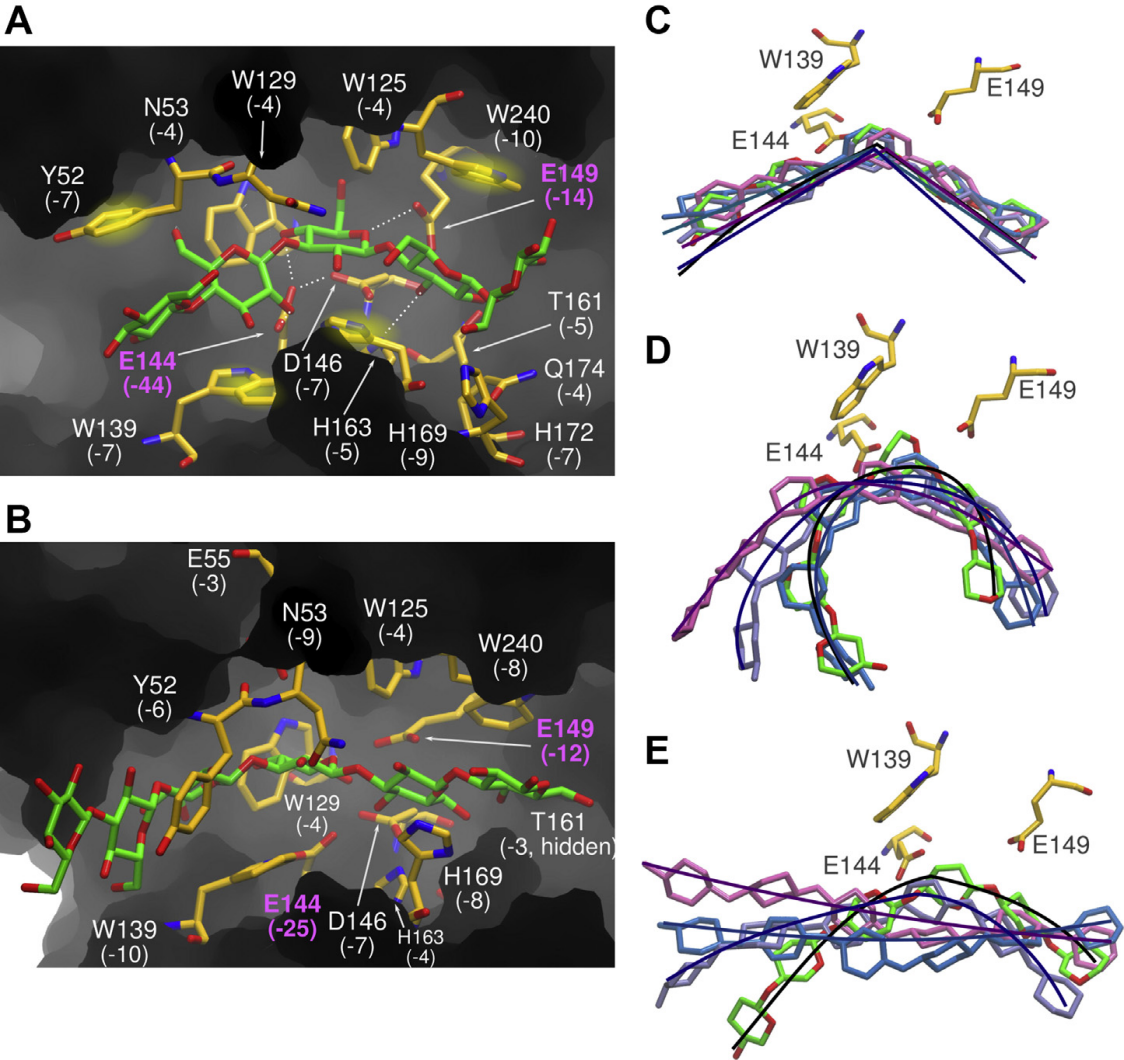
**Main interactions in the catalytic cleft with BG-2-1 (*A*) and L6 (*B*) observed in the molecular dynamics simulations**. The average energy of interaction computed in the simulations is noted in parenthesis (kilocalories per mole). Key residues harboring the substrate in the catalytic cleft via parallel stacking with the glycosyl rings are highlighted with a *bright yellow blur*. The most persistent hydrogen bond interactions are depicted by *dashed lines*. The other panels show the superimposition of the last frame of the triplicate simulations of the systems SCLam-BG-2-1 (*C*), SCLam-L6 (*D*), and SCLam-C6 (*E*) with their initial conformations. *Thick lines* along the oligosaccharide chains highlight their shape. The initial conformation of these ligands is colored in *green* and the simulation snapshots, in *ice-blue*, *mauve*, and *blue*.

The residues in the binding cleft that interact with the substrates are the same for all complexed structures. Stabilization is performed by hydrogen bonds with residues N53, E144, D146, E149, T161, H163, and H169 and CH-π interactions with Y52, W129, W139, and W240. (Fig. 6*B*). The interactions of SCLam^E144S^ with the ligands in each subsite are shown in Table 2.

**Table 2:** Ligand coordination of SCLam^E144S^ in each subsite

Subsite	H-bond	CH-π
−3		Y52
−2	N53 and H169	W139
−1	N53 and D146	W129
+1	T161, H163, and Q174^a^	W240
+2	Q174^a^	

a Hydrogen bond with glycosidic O between +1 and +2 glucoses existing only in SCLam^E144S^ with L6.

### Structural determinants for hydrolysis of β-1,3 and β-1,4 glycosidic bonds

Next, molecular dynamics (MD) simulations of SCLam bound to β-glucan oligosaccharides were conducted to identify persistent substrate interactions in the catalytic cleft. The first simulated systems were SCLam bound to (1,3,1,4)-β-glucopentaose, with a 1,3-β bond between −2 and −1 subsites (BG-2-1), and SCLam with L6. The simulations show that specific and dispersive interactions in the positive subsites, mostly at the +1 subsite, stabilize the glucose units (Glc), but the strongest interactions occur in the negative subsites. Simulations in triplicate of each system show consistency of the SCLam residues involved in the persistent interactions with the oligosaccharides (Fig. 6, *A* and *B*). A hydrogen bond network between the substrate and catalytic residues is found in the −1 and +1 subsites. The acid/base catalyst, E149, frequently forms hydrogen bond with O5 and O6 of the glucose unit at the −1 subsite. This carboxylic acid is also found close to the glycosidic bond oxygen, which is consistent with its role in favoring the initiation of the hydrolytic reaction.

In the MD simulations of SCLam-BG-2-1, the hydrogen bond interactions between the pairs E149–O5_Glc (subsite −1), E144–O4_Glc (subsite −1), E144–O2_Glc (subsite −1), and D146–E144 occur in about 36%, 29%, 16%, and 100% of the simulation time, respectively. E149 also interacts strongly with O3_Glc (subsite +1). In the simulation of SCLam-L6, the hydrogen bonds E149–O5_Glc (subsite −1), E149–O6_Glc (subsite −1), E144–O6_Glc (subsite −1), D146–O6_Glc (subsite −1), E144–D146, N53–Glc (subsite −2), and T161_Glc (subsite +1) occur during 22%, 14%, 26%, 11%, 100%, 30%, and 10% of the simulation time, respectively. The aromatic residues Y52, W139, and H169 at negative (−2/−1) subsites, and W240 at positive (+1/+2) subsites, are found persistently stacked over the pairs of glucose rings during the simulations. Although the aromatic ring of W129 is found stacked over the glucose ring at the subsite −1 in the initial configurations, this relative orientation does not persist over the course of the simulations. Several of these described specific and dispersive interactions are preserved relative to the crystal structure, although it is possible that the crystallographic arrangement is similar to an advanced step of the catalytic reaction mechanism or to the interaction between the enzyme and the reaction products. The change in relative orientation of the glucosyl unit in subsite −1, observed in the simulations, may reproduce the orientation of the substrate prior to any proton/electronic transfer in the catalytic site.

It is remarkable that the W129 residue drives the substrate chain conformation between −1 and +1 subsites. Superimposition of the last frames of the three simulations of SCLam-BG-2-1 (Fig. 6*C*) shows that the β-1,3 bond remains stable between the −2 and −1 subsites, with the substrate found in a hinge-like shape, which allows an optimum fit into the catalytic cleft of SCLam. The substrate containing only β-1,3 bonds, L6, is also found stable in the catalytic cleft, but it exhibits a U-shape, reflecting the helical conformation of β-1,3-glucans (Fig. 6*D*) (25, 26). The average energy interaction of BG-2-1 and L6 with the enzyme computed from the simulations is −142.1 and −119.3 kcal/mol, respectively, suggesting that the mixed β-1,3;1,4 bonds in BG-2-1 maximizes contact with the enzyme, supporting the molecular basis of the higher binding affinity of β-1,3-glucan compared with L6 that was observed in the ITC experiments.

In contrast, the simulation of (1,3,1,4)-β-glucopentaose, where the β-1,3 bond is located between the −1 and +1 subsites (BG-1+1), showed a rapid detachment of the glucose units at the −1 and +1 subsites. These data suggest that the position of the β-1,3 bond in a (1,3,1,4)-β-glucan is key in determining the hydrolysis efficiency.

The β-1,4 bonds of C6 are strained in the starting configuration of the simulations, and therefore the substrate is quickly displaced from the initial position to adopt a preferential linear conformation (Fig. 6*E*). Stacking interactions with aromatic residues maintain the substrate in the catalytic cleft during the simulations. Poor contact of C6 with the catalytic residues may impair the formation of the transition state and, thus, explain the low catalytic activity of SCLam toward cello-oligosaccharides.

### Insights of SCLam transglycosylation activity

Transglycosylation activity has been previously reported for members of the GH16 family (20). Replacement of the carboxylate nucleophile by a smaller residue, such as alanine or serine, and the addition of a modified substrate (typically α-glucosyl-fluoride) led to increased transglycosylation yields (7, 27, 28).

Transglycosylation was observed in SCLam (Fig. 2) and is supported by the crystallographic data obtained in this study. In all ligand-complexed structures, the bound substrates have a β-1,3 bond between moieties bound to the −1 and −2 subsites, even when the enzyme was incubated with a cello-oligosaccharide, which has only β-1,4 bonds. Moreover, the anomeric carbon of glucose in the subsite −1 is in the α-configuration. Therefore, the crystallographic structures can be used to study both the hydrolytic and the synthetic activities of the enzyme.

**Figure 7:**
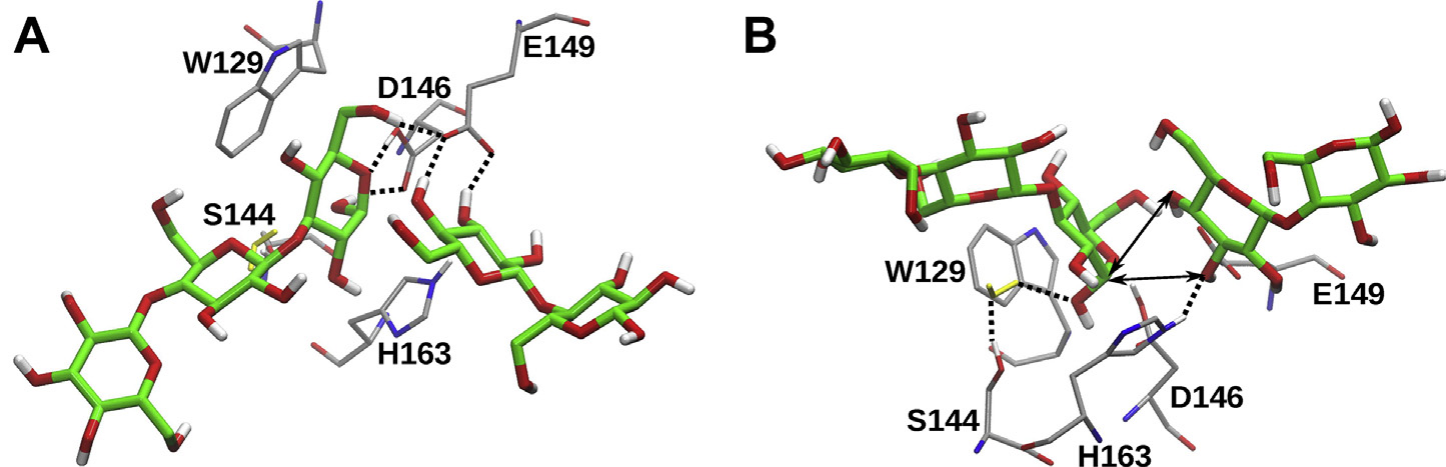
**Main hydrogen bond interactions in the catalytic cleft of SCLam**^**E144S**^**complexed to cellotriose and cellobiose in the negative and positive subsites, respectively, according to the molecular dynamics simulations**. *A* and *B* are two different views of a same representative simulation frame. The assigned interactions and their respective occurrence in the simulations are: E149–O3_Glc(+1), 53%; E149–O4_Glc(+1), 58%; E149–O6_Glc(−1), 51%; E146–O5_Glc(−1), 61%; E146–O1_Glc(−1), 15%; H163–O3_Glc(+1), 19%; water–O1_Glc(−1), 16%. A water molecule intermediating the interaction between Glc(−1) and S144 is depicted in *yellow*. The *black arrows* indicate the possible new glycosidic linkages (transglycosylation) between cellotriose and cellobiose due to the proximity of O3_Glc(+1) and O4_Glc(+1) to the anomeric carbon from Glc(−1).

We have performed MD simulations of crystallographic SCLam^E114S^ bound to both (1,3,1,4)-β-glucotriose and cellobiose in the negative and positive subsites, respectively. In this case, the acid catalyst E149 was set in its anionic form, thus reproducing the molecular state prior to transglycosylation. Of interest, unlike the simulations of SCLam-BG-2-1 and SCLam-L6, the position of the glucose unit in the −1 subsite is very stable in parallel orientation to the W129 aromatic group of SCLamE114S. The parallel orientation is stabilized because unprotonated E149 interacts only with 6-OH of the glucose unit at the −1 subsite, releasing the O5 of the pyranoside ring to interact with the auxiliary residue D146 (Fig. 7*A*). In contrast, the protonated E149, as a hydrogen bond donor, interacts both with 6-OH and O5 in the simulations of SCLam-BG-2-1 and SCLam-L6, described above (Fig. 6*A*). The protonation state of E149 affects not only the orientation of the glycosyl unit at the −1 subsite but also the stability of the cellobiose moiety at the positive subsites by forming hydrogen bonds with both 3-OH and 4-OH of the glucosyl unit at the +1 subsite. In contrast, in the three simulations of this system with protonated E149, the cellobiose totally escapes from the catalytic cleft.

Although the crystallographic structures suggested that a new glycosidic bond could be formed with the 3-OH group of the glycosyl unit at the +1 subsite, the position of the 4-OH group in the acceptor glycan is more favorable to a S_N_2 attack of the oxygen on the anomeric carbon at the −1 subsite (Fig. 7*B*). The average distances of 3-OH and 4-OH groups relative to the anomeric carbon are similar (4.0 ± 0.3 and 3.5 ± 0.3 Å, respectively), but the average angle O4...C1-O1 (166° ± 6°) is closer to the optimum value (180°) of nucleophilic substitution reactions than the angle O3...C1-O1 (125° ± 9°). Therefore, SCLam would be able to catalyze the formation of both β-1,3 and β-1,4 glycosidic bonds from (1,3,1,4)-β-glucotriose. Of interest, the experiments showed that both β-1,4 and β-1,3 bonds were produced when using L6 and C6 as the original substrates.

Collectively, the data presented herein elucidated details of the hydrolytic route of SCLam. In the general mechanism proposed for hydrolysis by GHs, proton transfer from the acid/base catalyst to the glycosidic bond is thought to occur in harmony with the nucleophilic attack of the anomeric carbon. Accordingly, our results suggest that the susceptibility to hydrolysis increases as the proton transfer occurs from E149 to the glycosidic bond, establishing the optimum orientation for reaction (*i.e.*, the glycosyl unit at the -1 subsite in parallel orientation relative to W129, Fig. 7*A*).

## Conclusion

SCLam is a (1,3/1,3;1,4)-β-D-glucan endohydrolase GH16 family member, with a low capacity to cleave β-1,4 bonds in cello-oligosaccharides. This behavior is shared by other enzymes of this group, such as LamR from *R. marinus* and TpLam from *T. petrophila*.

In the present study, the binding affinities of SCLam to several oligosaccharides, along with the crystallographic structure of the nucleophile mutant (SCLam^E144S^) bound to several ligands, provided detailed information about the configuration of the β-1,3 or β-1,4 glycosidic bonds in the catalytic pocket. MD simulations confirmed that the twisted β-1,3 glycosidic bond provides a favorable interaction with the binding site, which is partially achieved on linear β-1,4 glycosidic substrates.

## Experimental procedures

### Cloning, expression, and purification of SCLam and mutant SCLam^E144S^

The cloning and heterologous expression of SClam was performed as described (14). The mutant of SCLam, named SCLam^E144S^, resulted from the substitution of glutamic acid in position 144 for serine (E144S). The mutant form was obtained by using the Q5 Site-Directed Mutagenesis Kit (NEB) and following the manufacturer's instructions. In order to perform the protocol, two oligonucleotides were necessary: 5′-GGCACTGGGCtcAATCGACATCATGGAAATGGTCGC-3′ and 5′-GGCCAGCCGGTGCTGCCG-3′. By PCR, the construction containing the desired mutation (SCLam^E144S^-pET28a) was completely amplified. Next, the construction SCLam^E144S^-pET28a was transformed in *Escherichia coli* Rosetta2 strain (Novagen) and submitted to heterologous expression by growing the cells for 5 h at 37 °C and 180 RPM, followed by induction with 1 mM IPTG for 6 h at 20 °C. Protein purification consisted of affinity chromatography as described previously for the native SCLam, followed by dialysis in 20 mM sodium phosphate buffer, pH 6.5. The mutation was confirmed by Sanger sequencing analysis.

In both cases, the purity of protein samples obtained was confirmed by SDS-PAGE and the protein concentration was assessed by absorbance at 280 nm (molecular extinction coefficient for both wildtype and mutant: 82,975 M^−1^ cm^−1^).

### Capillary zone electrophoresis

The cleavage pattern of SCLam on laminarihexaose (L6), cellohexaose (C6), cellopentaose (C5), cellotetraose (C4), cellotetriose (C3), cellobiose (C2), 1,3-β-D-cellotriosyl-glucose (BGB), and 1,3-β-D-cellobiosyl-cellobiose (BGC) (all from Megazyme) was analyzed by capillary zone electrophoresis. The reactions were incubated at different times in 50 mM sodium phosphate buffer, pH 6.5, at 40 °C. The enzyme concentration varied according to the oligosaccharide compound used in the reaction: 4 μM for BGB and BGC, 0.5 μM for L6, 20 μM for C6, and 10 μM for C5, C4, C3, and C2. Cellopentaose, 5 mM, was incubated overnight with 10 μM of a purified preparation of laminarinase TpLam (GH16) from *T. petrophila* RKU-1 (Genbank Accession number ABQ46917.1) (17), at 70 °C. All the reactions contained 5 mM of the oligosaccharide.

After incubation, samples were labeled with 9-aminopyrene-1,4,6-trisulfonic acid by reductive amination derivatization (29), and hydrolysis products were evaluated by capillary electrophoresis (P/ACE MDQ system, Beckman Coulter) equipped with a laser-induced fluorescence detector. The separation occurred in a neutral capillary (Nano Separation Technologies) of 50 μm in internal diameter and 45 cm in length at 15 kV/70 to 100 μA in 40 mM potassium phosphate buffer (pH 2.5), with the cathode in the inlet. The retention times can vary slightly when comparing separate electrophoresis runs owing to the small volumes of capillary electrophoresis combined with small variations in the buffer. The electrophoretic behavior of oligosaccharide standards (Fig. S6) combined to coelectrophoresis was used to identify the products of enzyme action.

### High-performance anion exchange chromatography with pulsed amperometric detection

In order to evaluate the activity of SCLam with L6 and BGC in more detail, 200 μM of each substrate was incubated with 5 nM enzyme in 50 mM sodium phosphate buffer, pH 6.5, at 40 °C. Aliquots were taken after 2, 4, 8, 16, 40, 80, and 960 min, and the reactions were stopped at high temperature (95 °C) for 10 min. Samples (1 μl injections) were analyzed by the high-performance anion exchange chromatography system (ICS-5000, Dionex) equipped with a CarboPac PA-1 analytical column 4 × 250 mm with a CarboPac PA-1 guard (Dionex). Elutions were performed at 1.0 ml/min in 0 to 25 min, 100 mM NaOH; 25 to 40 min 100 mM NaOH with a 0- to 500-mM sodium acetate gradient. The data were analyzed using the Chromeleon Chromatography Data System.

### Isothermal titration calorimetry

Prior to ITC experiments, protein and ligands were dissolved in 20 mM sodium phosphate buffer, pH 6.5, in order to reduce the heat of dilution. Measurements were performed in a VP-ITC MicroCal (LNBIO/CNPEM) at 20 °C, with 1.4 ml of the 70 μM protein sample located in the reaction cell and 90 injections of 2 μl of ligand with 200-s intervals between each injection. The concentration of injected ligands varied from 1.5 to 10 mM, depending on the affinity of each one. The enthalpy values for ligands in the buffer were subtracted from the enthalpy values observed in protein–ligand reactions, followed by nonlinear regression analysis applying a single-site binding model. A sequential binding model was used to fit the data measured with ligands C6 and L6 (Origin, version 7.0). The thermodynamic parameters were calculated using the standard thermodynamic equation −RT ln*K*_a_ = *ΔG* = *ΔH* - *TΔS*.

### Crystallization and data collection

Samples of purified SCLam (in buffer: 20 mM Tris, pH 8.0, 150 mM NaCl, 1 mM DTT, 5% glycerol, 1 mM reduced L-Glutathione and 4% 2,5-hexanediol) and SCLam^E144S^ (in buffer: 20 mM sodium phosphate, pH 7.0) were concentrated to 17 and 10 mg/ml, respectively, and were used in the crystallization trials. For cocrystallization, the SCLam^E144S^ solution was supplemented with 1 mM of each ligand and incubated for 1 h at 4 °C. Initial screens were carried out using automated robotic systems Honey Bee 961 Dispensing System (DIGLABTM) (Molecular Biotechnology Group, Physics Institute of São Carlos) for SCLam, and HoneyBee 963 (LNBIO/CNPEM) for SCLam^E144S^, with commercial crystallization kits (Index, SaltRx and Crystal Screen, Hampton research; PACT and PEGs I and II Suite, Qiagen). A single SCLam crystal was obtained using the sitting-drop vapor diffusion technique at 18 °C, with drops containing equal volumes of protein sample and reservoir solution, comprising 2.1 M DL-Malic acid at pH 7.0. The diffraction data were collected on a Bruker APEX DUO single-crystal diffractometer system with KAPPA goniometer and an APEX II CCD detector (Molecular Biotechnology Group, Physics Institute of São Carlos). The data were integrated with the PROTEUM2 software (Bruker) and scaled using the program Aimless (30).

SCLam^E144S^ crystals were obtained using the hanging-drop vapor diffusion technique at 18 °C, with drops containing equal volumes (1 μl) of protein sample and reservoir solution. The reservoir solution contents where the SCLam^E144S^–ligand complexes were crystallized are described in Table S3. The diffraction data were collected via MX-2 beamline at the Brazilian Synchrotron Light Laboratory (LNLS-CNPEM), equipped with a Pilatus 2M detector. The data were integrated with the programs XDS (31) and iMosflm (32) and scaled with Aimless (30).

### Molecular replacement, model building, and structure refinement

The SCLam crystallographic structure was determined by molecular replacement (MR) with the program Phaser (33) using the coordinates of the laminarinase from *R. marinus* (Protein Data Bank [PDB] ID 3ILN) as a template. With 48% of sequence identity, the search model was manipulated with the Chainsaw program (Collaborative Computational Project No. 4 - Software for Macromolecular X-Ray Crystallography) prior to MR rotation and translation functions. SCLam^E144S^ structures were determined by MR using the coordinates of SCLam as a template.

Model building was carried out using the program Coot (34), and the refinements were performed by REFMAC5 (35). The stereochemical quality of the proteins and glycans was validated with the programs MolProbity (36) and Privateer (37), respectively. Visualization and all representations of the structures were carried out using the program PyMOL (The PyMOL Molecular Graphics System, Version 2.3.3, Schrödinger, LLC). The crystallographic structures were deposited in PDB with entries 6XOF, 6XQF, 6XQG, 6XQH, 6XQL, and 6XQM.

### Molecular dynamics

The initial structures used in MD simulations were generated using crystallographic structures of the enzymatic complexes obtained in this work or elsewhere (structural homologs) as templates. In the first part of this computational study, we examined the interactions of the enzyme with the oligosaccharides in a state preceding catalytic hydrolysis. For that, we performed MD simulations of SCLam bound to cellohexaose (C6), laminarihexaose (L6), and (1,3,1,4)-β-glucopentaose. The latter was constructed varying the position of the 1,3-β bond between the -1 and +1 subsites (BG-1+1) and between the −2 and −1 subsites (BG-2-1) in SCLam. The complexes SCLam-C6, SCLam-BG-2-1, and SCLam-BG-1+1 were prepared using the crystallographic structure of SCLam complexed with cellotriose in the negative subsites and cellobiose in the positive subsites. The glycosyl units were then connected to each other accordingly. To construct the SCLam-L6 complex, we used the crystallographic structure of the E115S laminarinase 16A from *Phanerochaete chrysosporium* complexed with laminariheptaose, L7, as a reference (PDB ID 2WLQ) (7). The ligand in this structure presents an open circular shape. Consequently, the reducing end occupies the −1 subsite, and the nonreducing end the +1 subsite. In order to have a continuous oligosaccharide in the catalytic cleft, we deleted the central glycosyl unit and connected the reducing and nonreducing ends of L7. In the second part of this MD study, we conduced simulations of the crystallographic complex of SCLam-E144S with cellotriose and cellobiose, which we hypothesize represents a configuration preceding transglycosylation, or equivalently the posthydrolysis state.

The protonation states of ionizable residues of SCLam were determined according to the pK_a_ values computed for pH 6.5, using the H++ server (38). Special attention was given to the protonation state of the catalytic triad. To reproduce a state preceding the retaining double-displacement substitution of the hydrolytic reaction (2), the nucleophile, E144, was set in its unprotonated form, while the auxiliary residue D146 and the acid/base catalyst, E149, were set in their protonated form. In simulations representing the posthydrolysis state, both protonated/unprotonated states of the acid/base catalyst were tested. Simulation boxes containing the enzymatic complex and 14,500 water molecules were built using Packmol (39), so that the hydration layers around the enzyme were at least 15 Å thick. A minimum of 50 Na^+^ and 50 Cl^−^ ions were also added. Ionic excess assured electrical neutrality, resulting in the ionic concentration of ∼0.16 M. The CHARMM27 force field was applied to the protein (40) and carbohydrates (41), and the TIP3P model was used for water molecules (42).

## Data availability

The data that support the findings of this study are available from the corresponding author upon reasonable request.

## Supporting information

This article contains supporting information.

## Conflict of interest

The authors declare that they have no conflicts of interest with the contents of this article.
