# Supplementary material for: Insights into the dual cleavage activity of the GH16 laminarinase enzyme class on β-1,3 and β-1,4 glycosidic bonds
Source: J Biol Chem. 2021 Feb 5;296:100385. doi: 10.1016/j.jbc.2021.100385 (PMC7961093; doi:10.1016/j.jbc.2021.100385)
Supplement: Supporting information [file mmc1.pdf]

Supporting Information

A structural basis of the cleavage mechanism on (1,3)- and (1,4)-β-D-glycosyl bonds of GH16 endo-β-1,3(4)-glucanase.

Marcelo Vizona Liberato[1,2,a], Erica Teixeira Prates[3,#,a], Thiago Augusto Gonçalves[2,4], Amanda Bernardes[5], Nathalia Vilela[2,4], Juliana Fattori[6], Gabriela Cristina Ematsu[1], Mariana Chinaglia[1], Emerson Rodrigo Machi Gomes[1], Ana Carolina Migliorini Figueira[6], André Damasio[4], Igor Polikarpov[5], Munir S. Skaf[3], Fabio Marcio Squina[2]*

[1] Centro Nacional de Pesquisa em Energia e Materiais (CNPEM), Laboratório Nacional de Ciência e Tecnologia do Bioetanol (CTBE), Campinas, São Paulo, Brasil

[2] Universidade de Sorocaba, Programa de Processo Tecnológicos e Ambientais, Sorocaba, SP, Brasil

[3] Universidade Estadual de Campinas (UNICAMP), Instituto de Química e Centro de Pesquisa em Engenharia e Ciências Computacionais, Campinas, SP, Brasil

[4] University of Campinas (UNICAMP), Department of Biochemistry and Tissue Biology, Institute of Biology, University of Campinas, Campinas, São Paulo, Brazil.

[5] Instituto de Física de São Carlos, Universidade de São Paulo, São Carlos, São Paulo, Brasil.

[6] Centro Nacional de Pesquisa em Energia e Materiais (CNPEM), Laboratório Nacional de Biociências (LNBio), Campinas, São Paulo, Brasil.

* Correspondence to fabio.squina@gmail.com ; Rod. Raposo Tavares, km, 92.5, Sorocaba-SP, CEP 18023-000, Brasil

[a] These authors contributed equally to this work.

[#] Present address: Biosciences Division, Oak Ridge National Laboratory, Oak Ridge, TN 37830, USA

Running title: Structural determinants of a GH16 endo-β-1,3(4)-glucanase

Keywords: glycoside hydrolase, transglycosylation, GH16, Laminarinase, endo-1,3(4)-β-glucanases, metagenome.

Table of Contents

1. Supplementary Methods

1.1 Molecular dynamics simulations

2. Supplementary Figures and Tables

Figure S1. Evaluation of the SCLam and TpLam cleavage pattern on cello-oligosaccharides

Figure S2. Breakdown and transglycosylation products of SCLam on laminarinohexaose and cellopentaose

Figure S3. Breakdown and transglycosylation products of SCLam ^{E144S} on 1,3-β- D-cellotriosyl-glucose (BGB), 1,3-β- D-cellobiosyl-cellobiose (BGC) and laminarinohexaose (L6).

Figure S4. Evaluation of the SCLam cleavage pattern on 1,3-β- D-cellobiosyl-cellobiose (BGC) and laminarinohexaose (L6).

Figure S5. SCLamE144S binding parameters measured by ITC.

Figure S6. Structural differences between SCLam and SCLam^{E144S}/C3 models.

Figure S7. Capillary zone electrophoresis of APTS-labeled laminarin-oligosaccharides, cello-oligosaccharides and glucose.

Table S1. Crystallographic data collection and refinement statistics.

Table S2. Amino acid residues that were not modelled due to poor electron densities.

Table S3. Reservoir solution contents used to co-crystallize SCLamE144S with each ligand.

Table S4. The relative activity of ScLam and SCLam^{E144S} on different substrates.

3. Supplementary References

1. Supplementary Methods

1.1. Molecular Dynamics Simulations

Three independent simulations of each system were performed according to the following protocol: (i) 5000 steps of conjugate gradient minimization (1) with the atomic positions of enzymatic complex and crystallographic water oxygens positions restrained; (ii) 500 steps of CG minimization with ligand and water atoms free to move; (iii) 500 steps of CG minimization with the whole system, except the α-carbons, unrestrained; (iv) 500 steps of CG minimization with all the atoms free to move; (v) gradual increasing of temperature and relaxation, comprising MD simulations of 100 ps at 283 K and 293 K; (vi) relaxation at the final temperature, 323 K, and production MD runs (50 ns). The preliminary simulations at 283 K of SCLam-C6 and SCLam-BGC (step v) was conducted restraining the positions of the glycosyl units at the positive subsites.

The MD simulations were performed under periodic conditions, using NAMD software (2). Langevin thermostat and Langevin piston Nosé-Hoover were used to control temperature and pressure, with 323 K and 1 bar as targets, respectively (3, 4). The electrostatic interactions were treated via the particle mesh Ewald method (5) and the short-range interactions were handled setting a 12 Å cutoff radius with smooth switching function. The RESPA multiple-time step algorithm (6) was employed with the shortest time step of 2 fs. All bonds involving hydrogen atoms were kept rigid using SHAKE (7).

2. Supplementary Figures and Tables

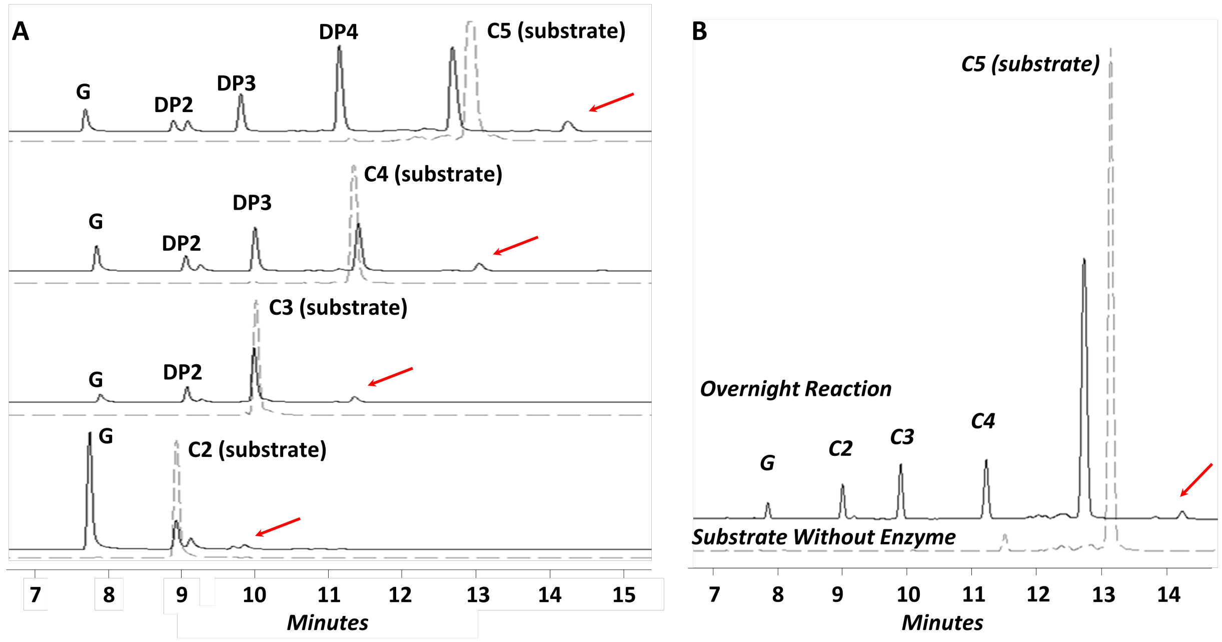

Figure S1. (A) Evaluation of the SCLam cleavage pattern on cello-oligosaccharides by Capillary Zone Electrophoresis (CZE), C5: cellopentaose; C4: celloheptaose; C3: cellotriose; C2: cellobiose. (B) Evaluation of the TpLam cleavage pattern from *Thermotoga petrophila* on C5. The reactions containing 5 mM of oligosaccharide and 20 µM of ScLam or 10 µM of TpLam were incubated overnight at 40 ºC. Separation was performed in a neutral capillary (Beckmam Counter) of 50 µm in internal diameter and 50 cm in length at 15 kV/70–100 µA in 40 mM potassium phosphate buffer (pH 2.5), with the cathode in the inlet. Dotted lines represent the substrate without the addition of enzyme.

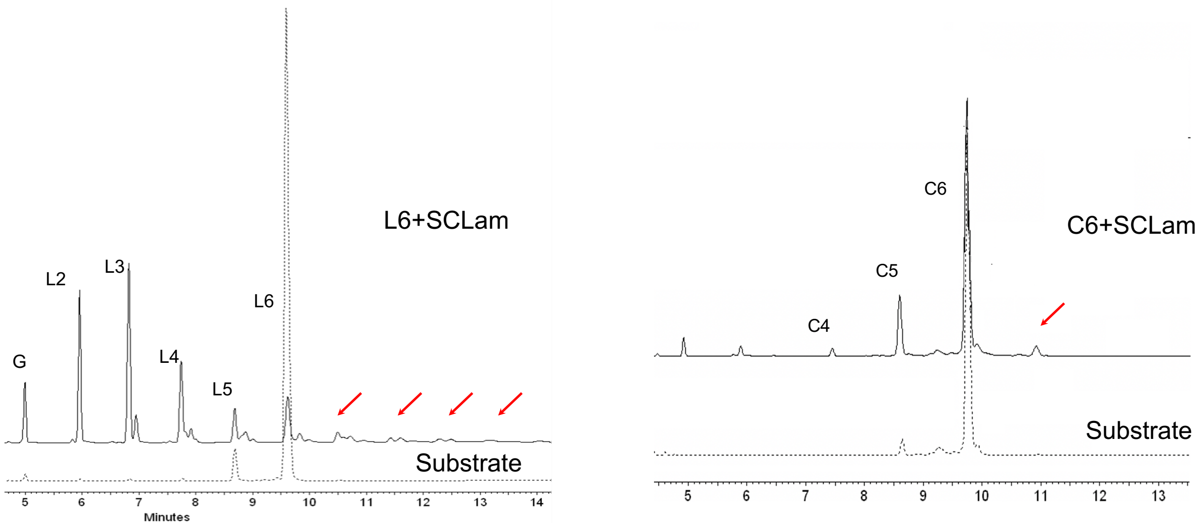

Figure S2. (A) Capillary zone electrophoresis of APTS-labeled breakdown and transglycosylation (arrows) products of ScLam on (A) laminarinohexaose and (B) cellohexaose. The reactions containing 5 mM of oligosaccharide and 0,5 µM and 20 µM of enzyme (A and B, respectively) were incubated at 40 ºC (15 minutes and 1-hour B, respectively). Separation was performed in a neutral capillary (Nano Separation Technologies-NST) of 50 µm in internal diameter and 45 cm in length at 70–100 µA in 50 mM potassium phosphate buffer (pH 2.5), with the cathode in the inlet.

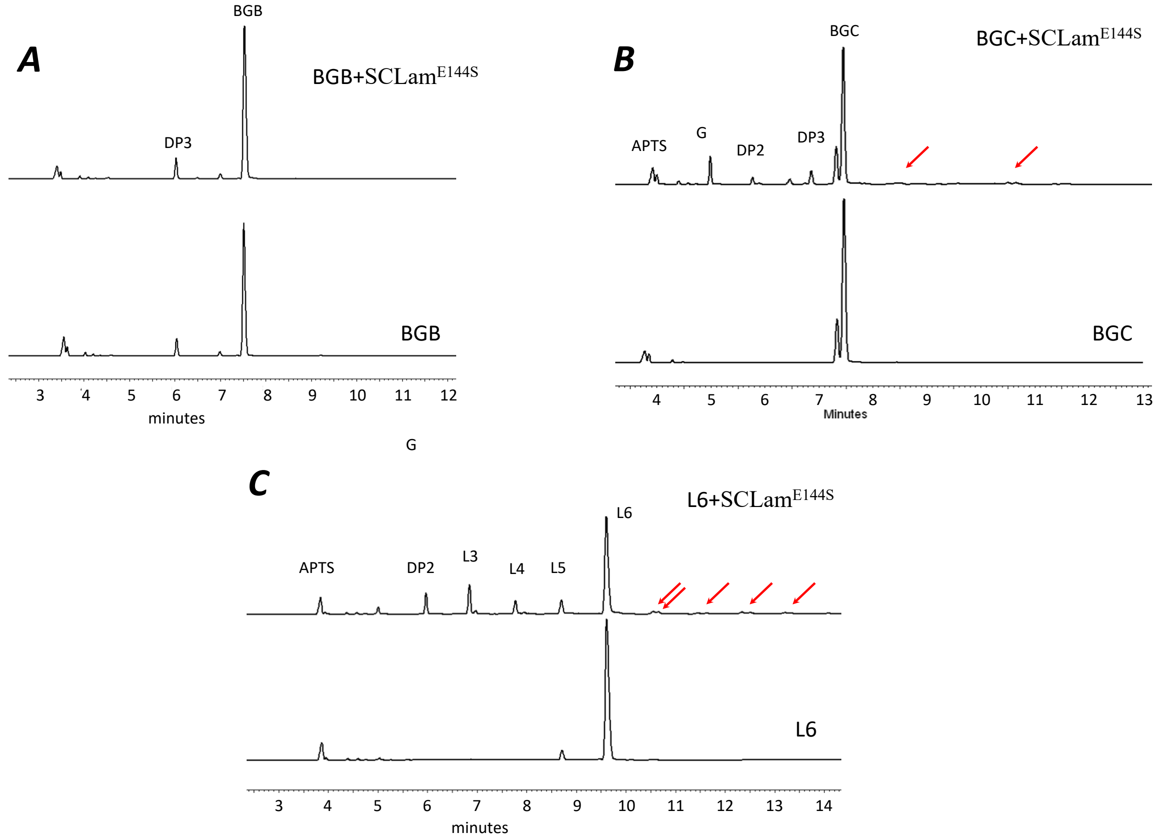

Figure S3. (A) Capillary zone electrophoresis of APTS-labeled breakdown and transglycosylation (arrows) products of ScLam ^{E144S} on (A) 1,3-β- D-cellotriosyl-glucose (BGB), (B) 1,3-β- D-cellobiosyl-cellobiose (BGC) and (C) laminarinohexaose. The reactions containing 5 mM of oligosaccharide and 4 µM, 4 µM and 0,5 µM of enzyme (A, B and C, respectively) were incubated overnight at 40 ºC. Separation was performed in a neutral capillary (Nano Separation Technologies-NST) of 50 µm in internal diameter and 45 cm in length at 70–100 µA in 50 mM potassium phosphate buffer (pH 2.5), with the cathode in the inlet.

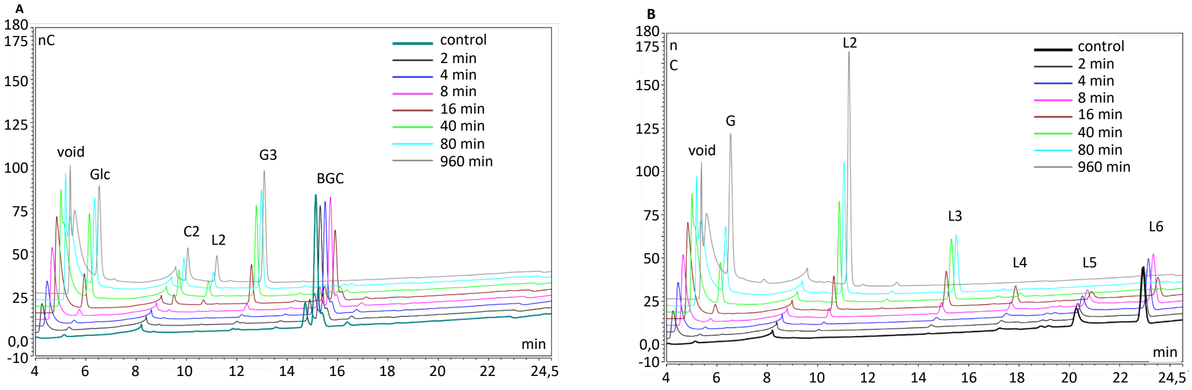

Figure S4. Evaluation of the SCLam cleavage pattern on (B) 1,3-β- D-cellobiosyl-cellobiose (BGC) and (C) laminarinohexaose (L6) by ion chromatography separation. Methods were described in the main manuscript.

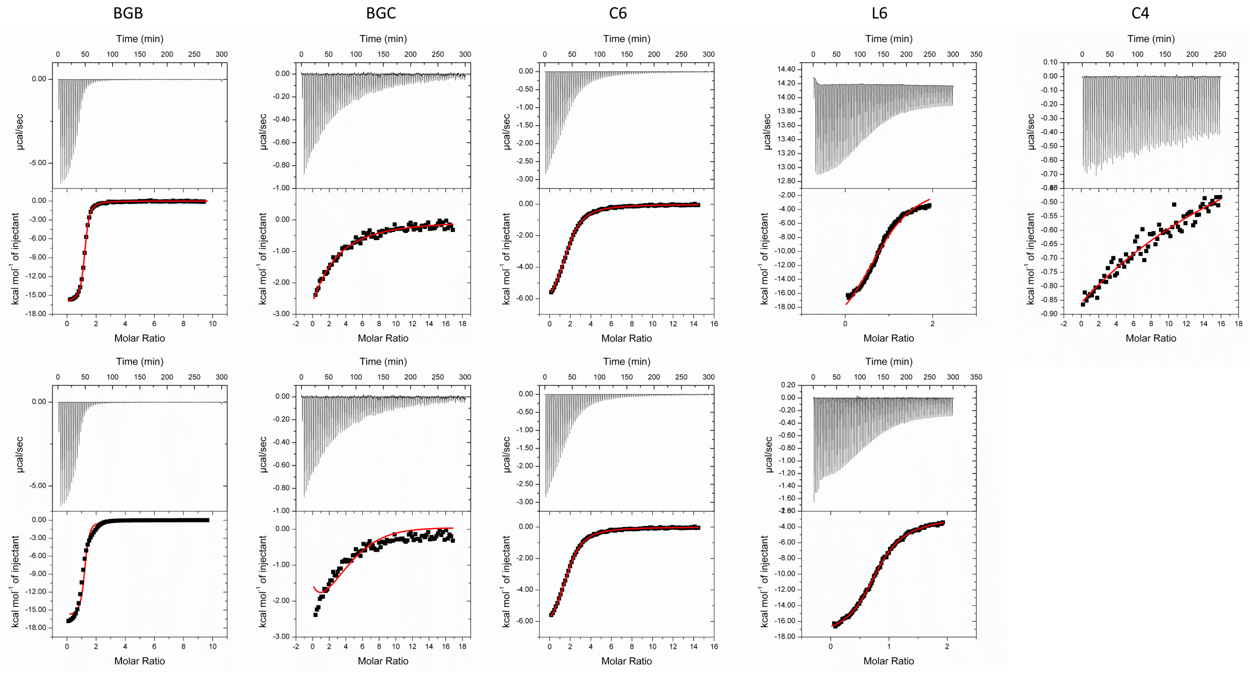

Figure S5. SCLam^{E144S} binding parameters measured by ITC. Data were measured titrating the ligands BGB, BGC, C6, L6, and C4 into mutated enzyme solution. The top graphs were fitted using single-site binding model, while a sequential binding model were used in bottom graphs. The top half of each panel shows the raw total heat of each reaction and the bottom half displays the resultant binding isotherms obtained by integrating the peak areas.

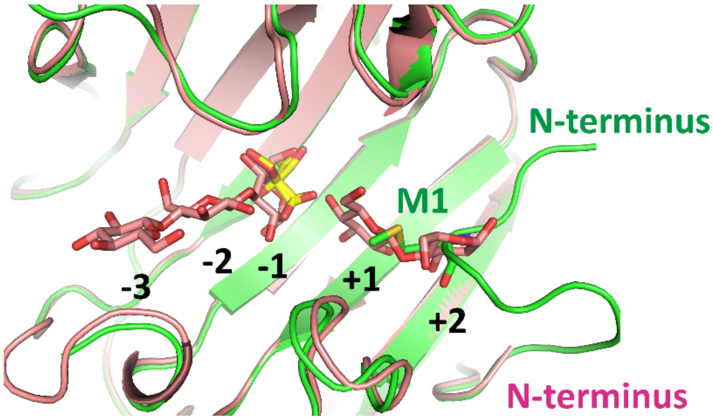

Figure S6. Differences between SCLam (green) and SCLam^{E144S}/C3 (pink) models assessed via structural superposition. SCLam has a glycerol molecule (yellow), modelled in three alternative conformations, bound to subsite -1. The N-terminal region is fitted to one end of the binding cleft, with the amino acid residue M1 occupying the subsites +1 and +2. Note that M1 is not the first amino acid of SCLam, there are another two (H-1 and S-2) that were added during cloning steps.

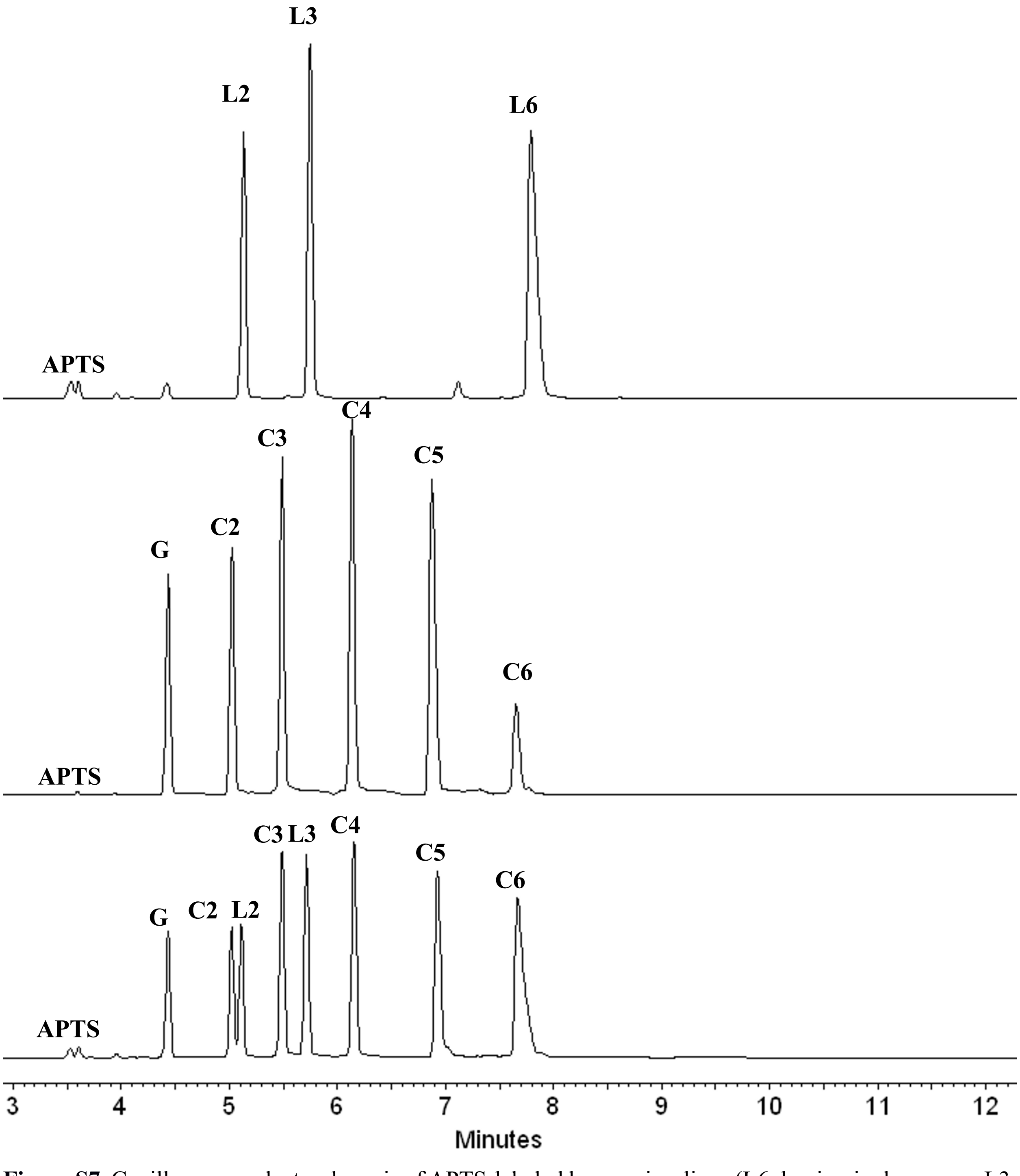

Figure S7. Capillary zone electrophoresis of APTS-labeled lamanarin-oligos (L6: laminarinohexaose -, L3: laminarinotriose and L2 laminarinobiose) cello-oligos (C6: cellohexaose, C5: cellopentaose, C4: celloheptaose, C3: cellotriose, C2: cellobiose and glucose (G).

Table S1. Crystallographic data collection and refinement statistics. Values in parenthesis refer to the outer shell.

	SCLam	SCLam E144S	SCLam E144S	SCLam E144S	SCLam E144S	SCLam E144S
Ligand	Apo	BGB	BGC	C3	C6	L6
**PDB id**	6XOF	6XQF	6XQG	6XQH	6XQL	6XQM
**Data collection**						
Wavelength (Å)	1.54	1.46	1.46	1.46	1.46	1.46
Space group	$P2_12_12_1$	$P2_12_12_1$	$P2_12_12_1$	$P2_12_12_1$	$P2_12_12_1$	$P2_12_12_1$
**Cell dimensions**						
a,b,c (Å)	42.9, 46.7, 115.1	38.7, 49.9, 114.5	38.7, 49.0, 114.3	38.7, 49.7, 115.3	38.4, 49.0, 114.0	40.2, 75.6, 83.5
Resolution (Å)	46.67-1.50 (1.53 - 1.5)	45.72-1.58 (1.61-1.58)	45.01-2.15 (2.22-2.15)	49.74-1.57 (1.60-1.57)	45.02-1.97 (2.02-1.97)	41.74-1.85 (1.89-1.85)
Mean I/σI	10.6 (1.4)	13.9 (2.4)	5.4 (1.6)	22.1 (4.3)	14.2 (2.8)	8.1 (1.3)
$CC_{(1/2)}$	1.00 (0.73)	0.99 (0.63)	0.97 (0.46)	0.99 (0.93)	0.99 (0.89)	0.99 (0.78)
Rpim	0.04 (0.49)	0.04 (0.25)	0.08 (0.42)	0.02 (0.14)	0.04 (0.21)	0.05 (0.45)
Completeness (%)	99.4 (97.2)	97.2 (77.7)	100 (100)	99.1 (82.3)	98.5 (92.8)	99.9 (99.4)
Redundancy	1.9 (1.8)	5.0 (2.9)	5.8 (5.3)	10.0 (5.3)	4.8 (3.2)	11.4 (9.6)
**Refinement**						
No. unique reflections	35738	28544	11788	30152	14777	21312
Rwork (%)	14.42	17.1	18.3	14.9	17.0	20.2
Rfree (%)	16.59	20.9	24.4	19.0	23.9	23.0
**No. atoms**						
Protein	2140	2039	1941	2081	2018	2090
Ligands/ions	19	35	35	58	35	41
Water	469	455	156	473	253	289
**Mean B-factor ($Å^2$)**						
Protein	8.8	10.4	29.1	8.5	23.8	26.2
Ligands/ions	4.1	9.4	29.6	11.1	22.5	23,8
Water	23.6	21.9	33.7	21.8	32.4	40.13
**R.m.s.d.**						
Bond (Å)	0.017	0.016	0.012	0.017	0.013	0.015
Angle (°)	2.08	1.94	1.83	2.03	1.90	1.87
**Ramachandran**						
Favoured (%)	97.70	95.31	95.49	94.57	95.72	93.49
Outliers (%)	0	0.39	0.41	0.39	0.39	1.15

Table S2. Amino acid residues that were not modelled due to poor electron densities.

Model	N-terminal	C-terminal
SCLamE144S/BGB	1-7	266
SCLamE144S/BGC	1-7	266
SCLamE144S/C3	1-6	-
SCLamE144S/C6	1-6	266
SCLamE144S/L6	1-3	-

Table S3. Reservoir solution contents that led to successful SCLamE144S co-crystallization with the ligands.

Ligand	Precipitant	Salt	Buffer
BGB	30 % PEG4000	0.2 MgCl	0.1 M Tris, pH 8.5
BGC	30 % PEG4000	0.1 MgCl	0.1 M Tris, pH 8.5
C3	27.5 % PEG4000	0.2 MgCl	0.1 M MES, pH 5.5
C6	22.5 % PEG4000	0.2 MgCl	0.1 M Tris, pH 8.5
L6	27.5 % PEG4000	0.2 MgCl	0.1 M MES, pH 5.5

Table S4. The relative activity of ScLam and ScLam^{E144S} on different substrates.

Substrate	ScLam	SCLam^{E144S}
barley β -glucan	100 %	1.3 %
laminarin	76.1 %	0.4 %
lichenin	45.4 %	2.1 %

The enzymatic reactions were incubated for 12 hours at 40 °C and pH 6.5. The reaction contained 0,5μM of ScLam or ScLam^{E144S} and the polysaccharides at 2μg.μl^{-1}. The biochemical properties of SCLam were comprehensive described in a previous work (8)

3. Supplementary References

1. Fletcher, R. (1964) Function minimization by conjugate gradients. *Comput. J.* **7**, 149–154
2. Phillips, J. C., Braun, R., Wang, W., Gumbart, J., Tajkhorshid, E., Villa, E., Chipot, C., Skeel, R. D., Kalé, L., and Schulten, K. (2005) Scalable molecular dynamics with NAMD. *J. Comput. Chem.* **26**, 1781–802
3. Schneider, T., and Stoll, E. (1978) Molecular-dynamics study of a three-dimensional one-component model for distortive phase transitions. *Phys. Rev. B*. **17**, 1302–1322
4. Martyna, G. J., Tobias, D. J., and Klein, M. L. (1994) Constant pressure molecular dynamics algorithms. *J. Chem. Phys.* **101**, 4177
5. Darden, T., York, D., and Pedersen, L. (1993) Particle mesh Ewald: An $N \cdot \log(N)$ method for Ewald sums in large systems. *J. Chem. Phys.* **98**, 10089–10092
6. Tuckerman, M., Berne, B. J., and Martyna, G. J. (1992) Reversible multiple time scale molecular dynamics. *J. Chem. Phys.* **97**, 1990
7. Ryckaert, J.-P., Ciccotti, G., and Berendsen, H. J. . (1977) Numerical integration of the cartesian equations of motion of a system with constraints: molecular dynamics of n-alkanes. *J. Comput. Phys.* **23**, 327–341

8 Alvarez, T. M., Liberato, M. V., Franco Cairo, J. P. L., Chinaglia, M., Polikarpov, I., Oliveira Neto, M., Squina, F. M. (2015) A NovelMember of GH16 Family Derived from Sugarcane Soil Metagenome Appl Biochem Biotechnol. **177**, 304–317.
